# Ecologically sustainable benchmarking of AI models for histopathology

**DOI:** 10.1038/s41746-024-01397-x

**Published:** 2024-12-24

**Authors:** Yu-Chia Lan, Martin Strauch, Pourya Pilva, Nikolas E. J. Schmitz, Alireza Vafaei Sadr, Leon Niggemeier, Huong Quynh Nguyen, David L. Hölscher, Tri Q. Nguyen, Jesper Kers, Roman D. Bülow, Peter Boor

**Affiliations:** 1 Institute of Pathology, University Clinic Aachen, RWTH Aachen University, Aachen, Germany; 2 Department of Public Health Sciences, College of Medicine, The Pennsylvania State University, Hershey, PA USA; 3 Department of Nephrology and Clinical Immunology, University Hospital Aachen, RWTH University Aachen, Aachen, Germany; 4 Department of Pathology, University Medical Centre Utrecht, Utrecht, The Netherlands; 5 Department of Pathology, Amsterdam UMC, University of Amsterdam, Amsterdam, The Netherlands; 6 Department of Pathology, Leiden Transplant Center, Leiden University Medical Center, Leiden, The Netherlands

**Keywords:** Medical research, Computational science

## Abstract

Deep learning (DL) holds great promise to improve medical diagnostics, including pathology. Current DL research mainly focuses on performance. DL implementation potentially leads to environmental consequences but approaches for assessment of both performance and carbon footprint are missing. Here, we explored an approach for developing DL for pathology, which considers both diagnostic performance and carbon footprint, calculated as CO_2_ or equivalent emissions (CO_2_eq). We evaluated various DL architectures used in computational pathology, including a large foundation model, across two diagnostic tasks of low and high complexity. We proposed a metric termed ‘environmentally sustainable performance’ (ESPer), which quantitatively integrates performance and operational CO_2_eq during training and inference. While some DL models showed comparable diagnostic performance, ESPer enabled prioritizing those with less carbon footprint. We also investigated how data reduction approaches can improve the ESPer of individual models. This study provides an approach facilitating the development of environmentally friendly, sustainable medical AI.

## Introduction

Many studies have shown the immense potential of deep learning (DL) to improve medical diagnostics in pathology^1–8^ and several DL models are already used in clinical routine^9–11^. Current developments largely focus on increasing the diagnostic accuracy (i.e., performance) of DL models. However, model training and inference can be computationally demanding, resulting in high electricity demands. This translates into high CO_2_ or equivalent emissions (CO_2_eq) depending on the country’s energy mix. Given the lack of renewable energies in most countries, increasing computational demands, and ongoing climate change, considerations towards more responsible and ecologically sustainable use of DL models in medicine are important. We previously calculated and modeled the carbon footprint of widespread DL model implementation in pathology^12^, which suggested considerable global warming potential. That study also suggested that ecological sustainability should be considered right from the beginning when the models are developed to mitigate long-term detrimental effects. However, approaches that would allow developing and benchmarking DL models not only for their performance but also their carbon footprint, were missing.

Here, we propose a framework for the development and benchmarking of DL models for computational pathology using the environmentally sustainable performance (ESPer) score. To develop ESPer, we tested four commonly used weakly supervised multiple instance learning models or models that can be used in a multiple instance learning (MIL) setting, and a novel pathology foundation model^13^ for two disease classification tasks of different complexity, i.e., kidney transplant pathology classification, where changes can be subtle and there is overlap between classes, and renal cell carcinoma subclassification, which has lower complexity due to strongly different morphology and mutual exclusivity. ESPer proved helpful in prioritizing models with lower CO_2_eq without loss of performance.

## Results

### Study outline

**Fig. 1:**
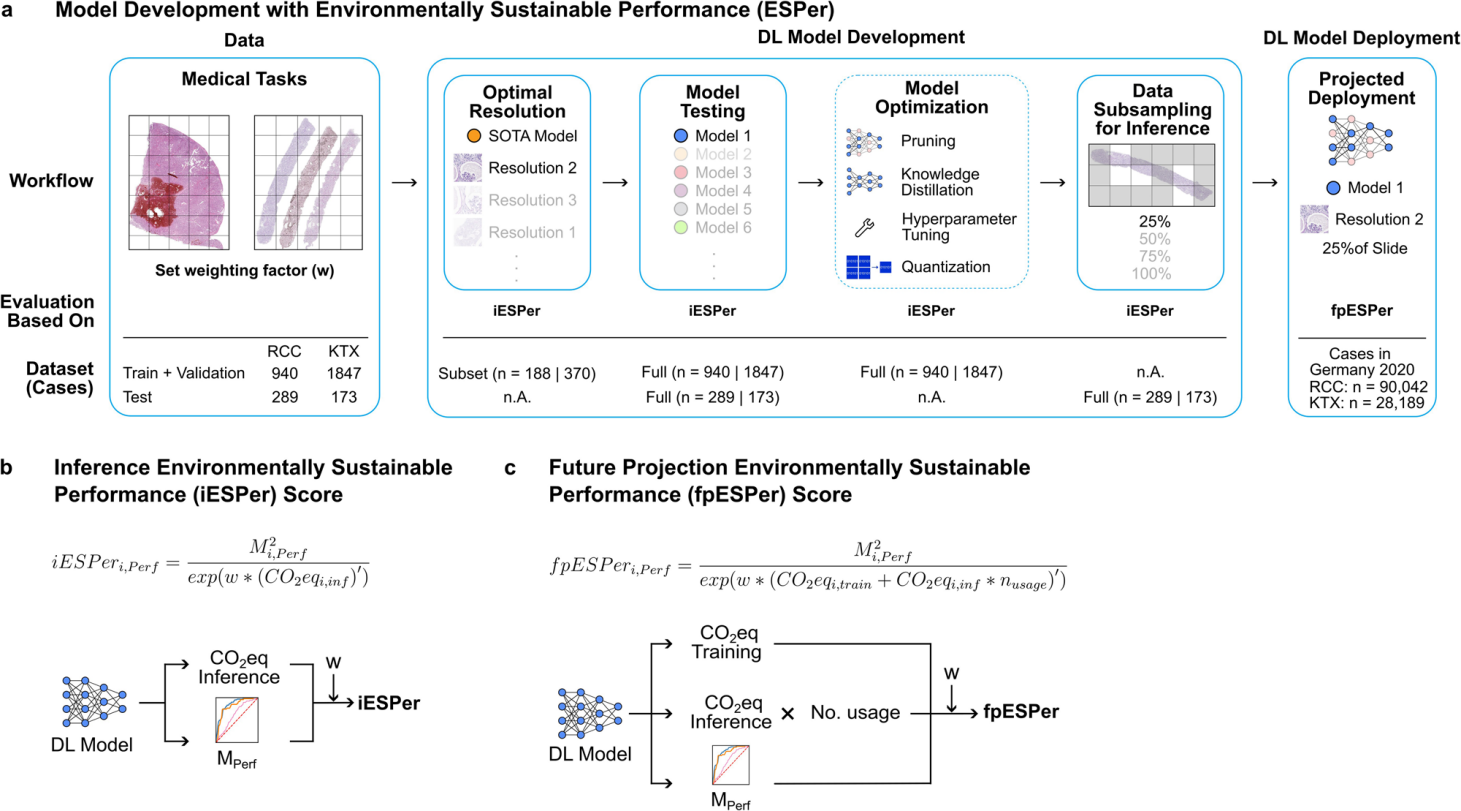
Model development with environmentally sustainable performance (ESPer) and ESPer scores. Study outline and environmentally sustainable performance (ESPer) metrics. **a** Workflow example for sustainable model development using the environmentally sustainable performance (ESPer) score (iESPer or fpESPer, see below). This includes various steps, ESPer metrics used for evaluation, and datasets. We used renal cell carcinoma subtyping (RCC) and kidney transplant disease classification (KTX) as use cases in our study. Based on the medical task, the weighting factor can be set upfront to prioritize between performance and ecological sustainability. The dataset row indicates which amount of data needs to be used for each step of model development. There are various approaches for model optimization, such as pruning, knowledge distillation, hyperparameter tuning, or quantization. These were described before and not tested here but were included in the figure to provide a more complete picture of model development. **b** Formula and a diagram for the inference environmentally sustainable performance (iESPer), where ${\rm{iESPe}}{{\rm{r}}}_{{\rm{i}},{\rm{Perf}}}$ is the iESPer score for model ${\rm{i}}$ in the comparison series and performance metric ${\rm{Perf}}$, ${{\rm{M}}}_{{\rm{i}},{\rm{Perf}}}$ is the measured metric for model ${\rm{i}}$, $w$ is the weighting factor, ${{\rm{CO}}}_{2}{{\rm{eq}}}_{{\rm{i}},\inf }$ is the CO_2_eq produced by model ${\rm{i}}$ during inference and ${\rm{X}}^{\prime}$ is the range normalization operation for ${\rm{X}}$. **c** Formula and diagram for the future projection ESPer (fpESPer). The notation is similar to the formula in (**b**), with the addition that, ${{\rm{CO}}}_{2}{{\rm{eq}}}_{{\rm{i}},{\rm{train}}}$ is the CO_2_eq produced by model ${\rm{i}}$ during training and that ${{\rm{n}}}_{{\rm{usage}}}$ is the projected number of usages for the model.

We used five datasets for two clinically relevant tasks in pathology, i.e., the classification of renal cell carcinoma (RCC) subtypes (*n* = 1229 cases) and kidney transplant (KTX) diseases (*n* = 2020 cases). Further details on the datasets are shown in Supplementary Fig. 5. We selected four different approaches that are currently commonly used in computational pathology for the classification of whole slide images (WSI), i.e., transformer-based correlated multiple instance learning (TransMIL)^14^, clustering-constrained attention multiple instance learning (CLAM)^15,16^ InceptionV3, a vision transformer (ViT)^17^, and providence whole-slide pathology foundation model (Prov-GigaPath)^13^. We calculated diagnostic accuracy, including validation on external unseen cohorts, and carbon footprint, and integrated both in a novel metric (Fig. 1b, c).

### Diagnostic performance of the models

**Fig. 2:**
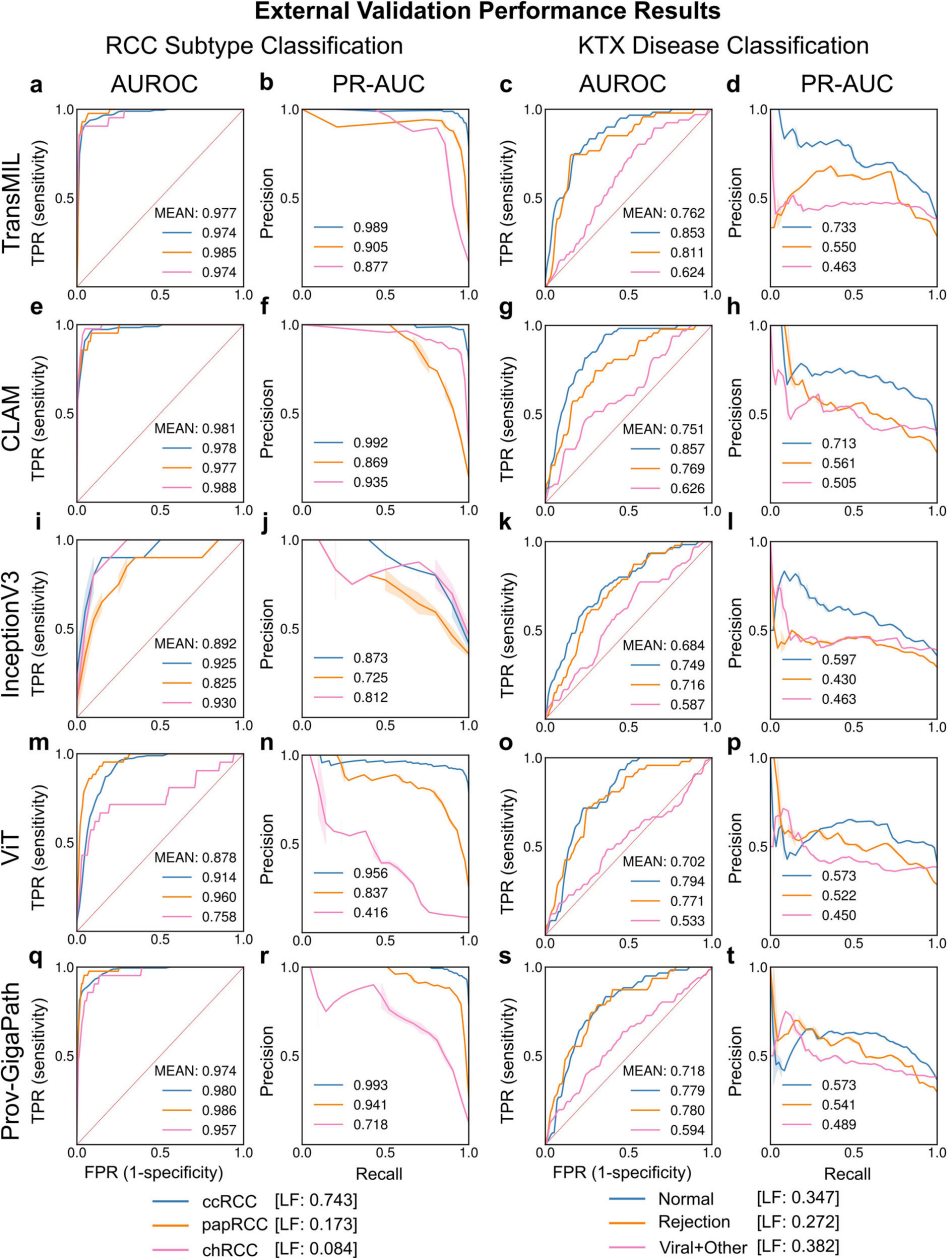
Performance results for RCC-subtype classification and KTX disease classification on external validation set. Performance results of TransMIL, CLAM, InceptionV3,ViT, and Prov-GigaPath models for RCC subtype (*n* = 289) and KTX disease classification (*n* = 173) tasks. **a**, **e**, **i**, **m**, **q** show the AUROC and **b**, **f**, **j**, **n**, **r** show the AUPRC for all models for the RCC-subtype classification task, including Prov-GigaPath. **c**, **g**, **k**, **o**, **s** show the AUROC and **d**, **h**, **l**, **p**, **t** show the AUPRC for all models for the KTX disease classification task, respectively. LF label frequency of the corresponding class, TPR true positive rate, FPR false positive rate, AUROC area under the receiver operating characteristics curve, PR-AUC precision-recall area under the curve, RCC renal cell carcinoma, ccRCC clear cell renal cell carcinoma, papRCC papillary renal cell carcinoma, chRCC chromophobe renal cell carcinoma.

CLAM showed the best performance measured as mean area under the receiver operating characteristics curve (AUROC) in the RCC-subtype classification task (0.984 [95%-CI: 0.968–0.995]), while TransMIL achieved the highest mean AUROC in the KTX disease classification task (0.763 [95%-CI: 0.707–0.815]) in the external unseen cohorts (Fig. 2a–g). CLAM showed the highest performances in the training validation datasets (Supplementary Fig. 1a–p). Supporting the diagnostic model performance, gradient-weighted class activation maps (gradCAM) revealed areas showing typical morphology for the respective RCC subtypes (Supplementary Fig. 2a–i). Similarly, gradCAM highlighted diagnostically relevant areas in the kidney transplant disease classification task (Supplementary Fig. 3a–i).

The performance and generalizability of all models were lower in the more challenging classification of kidney transplants compared to RCC subtypes (Fig. 2a–o and Supplementary Fig. 1). One of the most challenging tasks is to differentiate between rejection and viral disease in transplant, particularly without ancillary studies, which was reflected by the weak performance on the KTX-classification task for the class “Viral + Other”.

### CO_2_eq emissions of the models

**Fig. 3:**
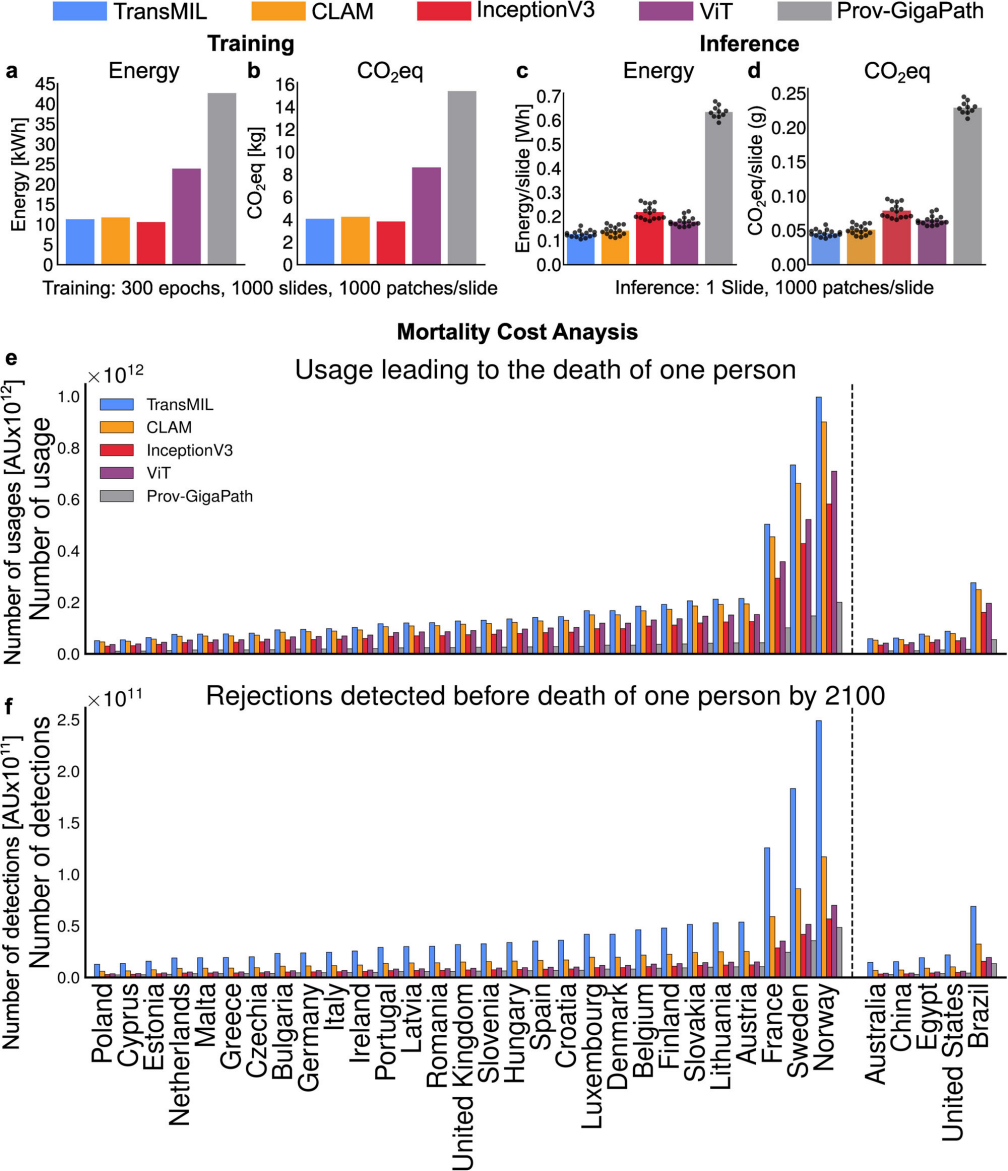
Environmental impact of training and inference. Training and inference impact the environment on different scales. Due to the quantity of data needed for training and backpropagation being disabled for model inference, an inference run consumes much less energy than training a model. **a**, **b** show the energy consumption and CO_2_eq footprint for training (*n* = 1) and **c**, **d** show the mean energy consumption (*n* = 15) and CO_2_eq footprint for inference for each evaluated model, respectively. **e** shows the number of usage of a model in a certain country, before one temperature related excess death occurs because of the emitted CO_2_eq, calculated on the basis of the mortality cost of carbon^18^. Based on the number of usage, the number of positive predictions are shown in (**f**). Panels **e**, **f** were calculated based on the mean CO_2_eq of (**d**). AU arbitrary units, KTX kidney transplant.

In both tasks, TransMIL and CLAM showed the lowest CO_2_eq emissions during training (Fig. 3a, b), while the ViT and the InceptionV3 showed more than 600 times higher CO_2_eq emissions (Fig. 3a, b and Table 1). During inference, CO_2_eq emissions of CLAM were slightly higher compared to TransMIL (0.048 g [95%-CI: 0.044–0.051] and 0.046 g [95%-CI: 0.043–0.049] respectively, Fig. 3c, d and Table 1). InceptionV3 produced more CO_2_eq emissions than the ViT during inference (0.073 g [95%-CI: 0.069–0.076] and 0.062 g [95%-CI: 0.059–0.064] respectively, Fig. 3c, d and Table 1).

**Table 1:** Energy consumption and CO_2_eq emission for model training and inference

	Training		Inference		
Model	Energy (kWh)	CO_2_eq (kg)		Energy (Wh)	CO_2_eq/Slide (g)
TransMIL	11.263	4.065	0.128		0.046
CLAM	11.713	4.228	0.132		0.048
InceptionV3	10.584	3.821	0.201		0.073
ViT	23.873	8.618	0.170		0.065
Prov-GigaPath	42.625	15.388	0.63		0.229

Energy consumption and CO_2_eq emission for model training and inference for the evaluated models TransMIL, CLAM, InceptionV3, ViT, and Prov-GigaPath. The CO_2_eq is calculated from the energy measured directly from low-level APIs multiplied by the local values for carbon intensity (Germany). For training, it was assumed that each model would be trained once in the time period, for 300 epochs, with a dataset with 1000 WSIs with 1000 patches, each with resolutions of 224px/256μm. CO_2_eq for inference was measured on one WSI with 1000 patches and of patch resolution of 224px/256μm.

Additionally, we evaluated the mortality cost when using a DL model due to the CO_2_eq emissions. This was based on the previously published mortality cost of carbon^18^, estimating that every 4400 Tons of CO_2_eq added to the 2020 baseline will result in one temperature-related excess death globally by 2100. Setting this estimate as a threshold, countries with a high share of renewable energy are able to produce more electricity for every 4400 Tons of CO_2_eq. Conversely, these countries are able to run model inferences more often before the CO_2_eq emissions result in one excess casualty according to the mortality cost. The most inferences, until one excess death due to CO_2_eq emissions occurs, could be performed using TransMIL in countries with a high share of renewable energy, such as Norway (Fig. 3g). Higher number of model inference naturally results in higher impact of using such deep learning models. Although the ranking of the models does not change depending on the country, the local energy mix significantly impacts the actual CO2eq emissions.

Due to model inference being required to generate heatmap visualizations, each GradCAM visualization produces approximately 0.013 g CO_2_eq per slide in our setting.

### Environmentally sustainable performance (ESPer) score

**Fig. 4:**
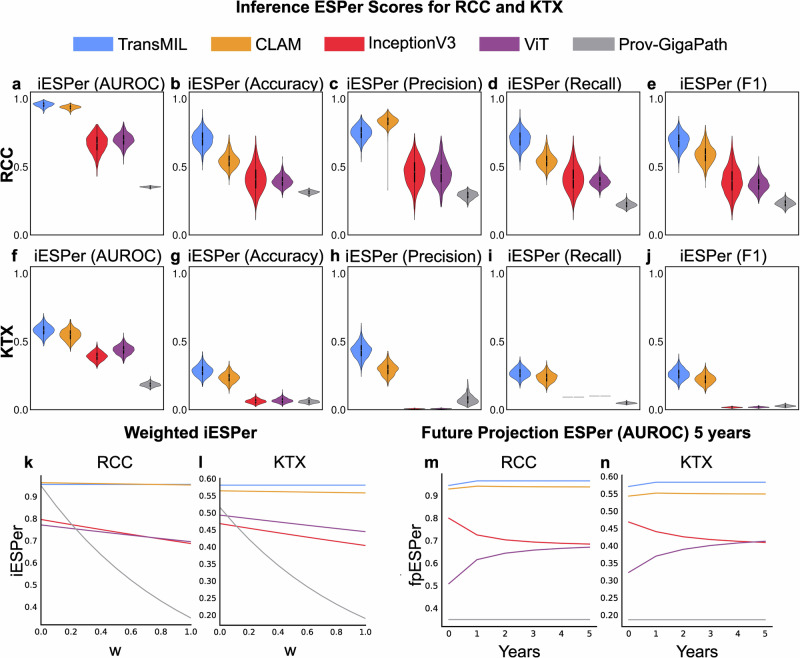
ESPer scores. **a**–**e** show the ESPer scores calculated for the performance metrics AUROC accuracy, precision, recall, and F1, for all investigated models for RCC (*N* = 289). **f**–**j** show the same for KTX (*N* = 173). **k**, **l** show how changing the weighting factor $w$ impacts the ranking of iESPer scores for RCC and KTX, respectively. **m** shows the projection of ESPer scores calculated from the AUROC metric for 5 years, based on the number of RCC cases in the EU for 2019 (*n* = 90,042). **n** shows the same, based on the number of kidney transplant cases in the EU for 2019 (*n* = 28,189). All CO_2_eq emissions in this figure are based on the energy mix of Germany. AUROC area under the receiver operating characteristics curve, iESPer inference environmentally sustainable performance, fpESPer future projection environmentally sustainable performance, RCC renal cell carcinoma, KTX kidney transplant.

ESPer scores enable a quantitative assessment of model performance and its carbon footprint. iESPer integrates a performance metric and CO_2_eq emissions of tested models for a given task at a single inference (Fig. 1b). Training and Inference ESPer can also be integrated over time, e.g., when used in clinical practice (Future Projection ESPer - fpESPer, Fig. 1c). The fpESPer score is calculated based on the training CO_2_eq emissions of all models. ESPer scores can be calculated using the different performance metrics, i.e., AUROC, accuracy, precision, recall, or F1-index (Tables 2, 3). Apart from the iESPer for precision, where CLAM scored highest, TransMIL had the highest iESPer scores in both tasks and across all performance metrics, with CLAM having the second highest iESPer scores (Table 2 and Fig. 4c–l). We chose the most commonly used performance metric, AUROC to assess the fpESPer.

**Table 2:** iESPer scores for RCC and KTX

RCC Task (iESPer)											
MODEL	CO2eq/Slide (g)	AUROC	95%CI	ACCURACY	95%CI	PRECISION	95%CI	RECALL	95%CI	F1	95%CI
TransMIL	0.046	0.964	0.936–0.986	0.8	0.732–0.868	0.736	0.634–0.835	0.695	0.577–0.822	0.676	0.556–0.798
CLAM	0.048	0.937	0.908–0.960	0.815	0.753–0.874	0.833	0.764–0.897	0.54	0.442–0.661	0.583	0.453–0.717
InceptionV3	0.073	0.665	0.511–0.788	0.41	0.238–0.580	0.451	0.258–0.640	0.41	0.234–0.592	0.394	0.218–0.584
ViT	0.065	0.693	0.593–0.784	0.643	0.571–0.718	0.448	0.270–0.654	0.393	0.316–0.485	0.37	0.272–0.484
Prov-GigaPath	0.229	0.349	0.336–0.360	0.303	0.279–0.325	0.261	0.206–0.318	0.214	0.177–0.259	0.219	0.175–0.267
**KTX Task (iESPer)**											
MODEL	CO2eq/Slide (g)	AUROC	95%CI	ACCURACY	95%CI	PRECISION	95%CI	RECALL	95%CI	F1	95%CI
TransMIL	0.046	0.579	0.501–0.660	0.279	0.209–0.368	0.428	0.322–0.533	0.265	0.200–0.345	0.257	0.184–0.343
CLAM	0.048	0.547	0.472–0.627	0.234	0.169–0.312	0.294	0.216–0.385	0.236	0.171–0.306	0.223	0.157–0.295
InceptionV3	0.073	0.391	0.334–0.451	0.062	0.038–0.097	0.007	0.004–0.011	0.093	0.093–0.093	0.017	0.012–0.024
ViT	0.065	0.439	0.373–0.511	0.068	0.037–0.105	0.008	0.004–0.012	0.1	0.100–0.100	0.019	0.011–0.026
Prov-GigaPath	0.229	0.188	0.160–0.222	0.065	0.044–0.089	0.110	0.062–0.168	0.055	0.041–0.072	0.042	0.025–0.062

Mean iESPer scores for each metric with corresponding confidence intervals (95%), for each examined model benchmarked on the RCC and KTX task, including CO2eq measurements per slide for inference, respectively.

For RCC tasks, TransMIL has the highest fpESPer from the beginning. InceptionV3 starts with a higher fpESPer, but ViT nearly matches the score after 5 years (Fig. 4m).

For the KTX task, while InceptionV3 initially has a better fpESPer score than ViT, after four years of inference, ViT shows the same fpESPer as InceptionV3, and has a higher fpESPer after that (Fig. 4n), thereby representing a more sustainable model. In the RCC task, TransMIL has the highest fpESPer from the beginning (Fig. 4n). Prov-GigaPath consistently performs worse due to the high combined CO_2_eq emissions, despite good performances in both tasks.

For some tasks, different weighing between performance and CO_2_eq emissions might be required. One way to manually introduce the weighting into the ESPer score is to multiply the normalization term with a weighting factor w, with w∈[0,1]. Setting $w$ to zero would completely “ignore” the impact of CO_2_eq on ESPer, thereby only reflecting performance. The setting of the $w$ value is highly dependent on the application scenario and allows individual setting by the experts and researchers for each specific task. The effect of choosing different weighting factors is shown in Fig. 4k, l.

### Reduction strategies improving ESPer

**Fig. 5:**
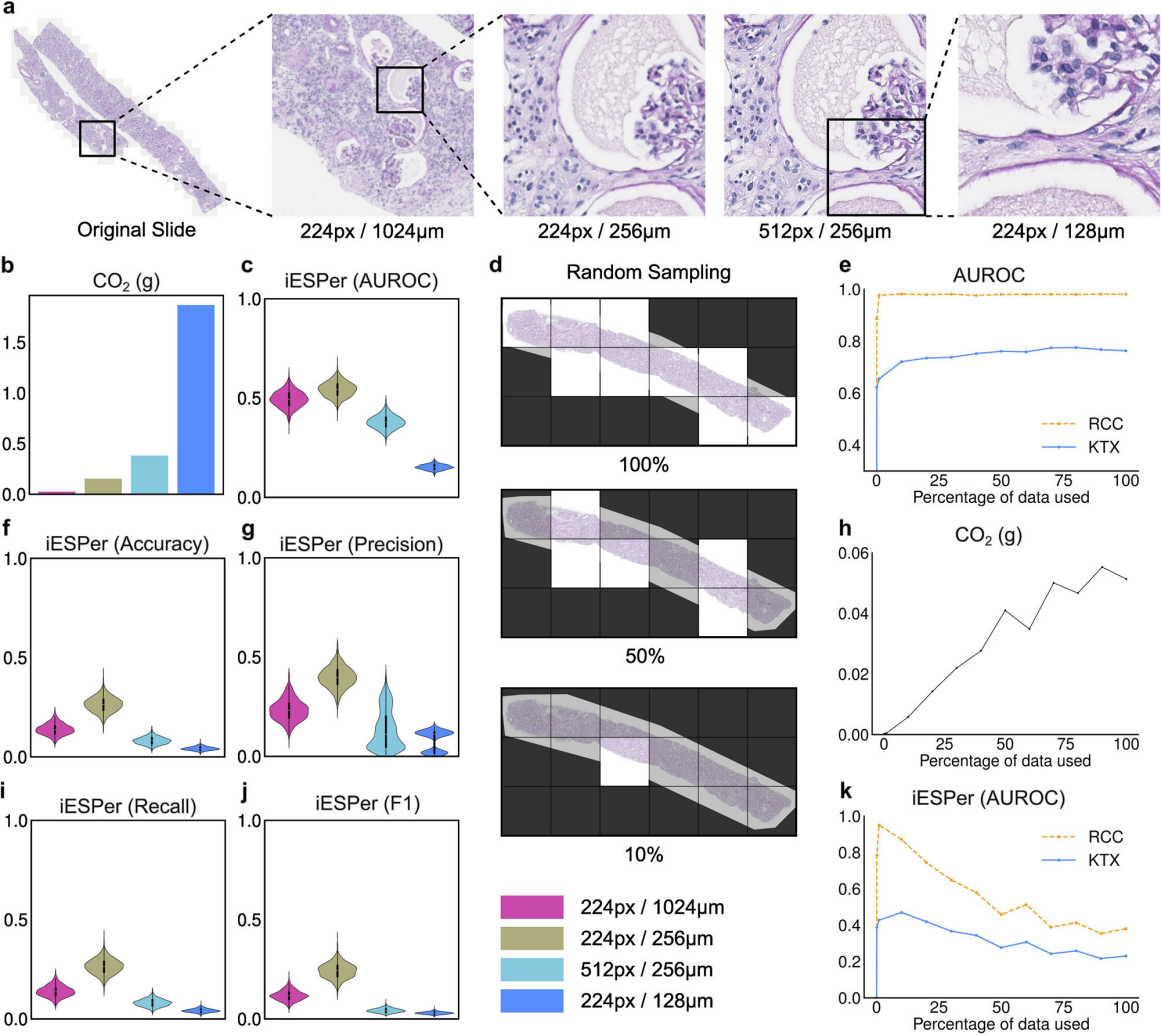
Reduction strategies. **a** Presents examples of different image patches with different sizes and resolutions from the same slide. The following measurements and calculations were performed for TransMIL. **b** Shows the CO_2_eq emission for model inference per slide (*n* = 15) and **c**, **f**, **g**, **i**, **j** Show the iESPers calculated for each image size and resolution pairing for the KTX task (*n* = 173). **d** Demonstrates random sampling and reduction of tiles, while **e** shows the AUROC progression from using 100% available patches to 10% of patches. **h** Shows the corresponding CO_2_eq emissions and **k** shows the iESPer scores for each percentile measured for the tasks RCC (*n* = 289) and KTX (*n* = 173). AUROC area under the receiver operating characteristics curve, iESPer inference environmental sustainable performance, RCC renal cell carcinoma, KTX kidney transplant.

Approaches for reducing the carbon footprint of models while retaining diagnostic performance would be highly desirable for sustainable and widespread use. Here, we investigated two data reduction strategies for this, i.e., using tiles of different sizes and resolutions and reducing the number of tiles used per WSI. We used the architecture with the best iESPer, i.e., TransMIL (Fig. 5a).

The highest CO_2_eq emissions per case were produced using the smallest physical tiles (corresponding to edge lengths of 128 µm) this is likely because the number of tiles per case increases with a smaller area per tile, while the image size (number of pixels) for each tile remains the same (resizing to 224 × 224 pixels, Fig. 5b). This translates to the lowest iESPer score of the investigated tile sizes (Fig. 5). The lowest CO_2_eq emissions were produced using tiles with edge lengths of 1024 µm on 224 pixels. However, this resolution did not result in the highest iESPer scores, mainly because of low model performance. We hypothesize, that this is due to the low level of critical structural detail at this resolution. The best configuration resulting in the highest iESPer score was a tile edge length of 256 µm on 224 pixels (Table 3 and Fig. 5).

**Table 3:** iESPer for different resolutions in the KTX task

Resolution	CO_2_eq/Slide (g)	AUROC	iESPer (AUROC)	95%CI
224px/1024 μm	0.025	0.702	0.493	0.410–0.581
224px/256 μm	0.138	0.762	0.542	0.468–0.613
512px/256 μm	0.383	0.678	0.377	0.317–0.442
224px/128 μm	1.87	0.640	0.151	0.124–0.179

iESPer scores and 95% confidence intervals for the AUROC metric for different resolutions for TransMIL.

Previous works suggested that testing the efficacy of the data reduction strategies is sufficient when performed on a representative subset of the training and validation dataset^19,20^. This could reduce CO_2_eq emissions during model development. Additionally, early stopping can be employed to reduce the number of epochs trained. A comparison of iESPer scores for different training epochs is shown in Supplementary Table 2. In this study, we used a fixed number of training epochs for all models to allow better comparability. For the RCC-subtype classification, only a fraction (10%) of the WSI was sufficient to reach an AUROC comparable to using 100% of the tiles (Fig. 5e). This is likely due to the rather homogeneous growth pattern and morphological appearance of the analyzed RCC subtypes. However, a similar trend was observed for the more complex kidney transplant disease classification. This is surprising since kidney rejection and some other diseases can be very focal, with defining lesions only appearing in small areas of the biopsy.

CO_2_eq emissions increase progressively with the share of tiles used. With a plateauing AUROC, tracking the iESPer for the number of tiles allows to find the optimal fraction of tiles to maintain performance while decreasing the CO_2_eq footprint.

In total, ~77.8 kg CO_2_eq were produced to generate the results in this work.

## Discussion

The majority of studies describing the development of novel DL models for pathology focused on diagnostic accuracy. The ecological consequences of training and using these models have been largely neglected, potentially due to the lack of data on real-world workflows and metrics enabling quantitative assessment during the development and testing of DL models. In addition, commercial DL applications are often offered as cloud solutions, and to our knowledge, no data on the carbon footprint of such services are publicly available. While the energy consumption and CO_2_eq emissions of a single DL model inference are usually numerically low, the amount and size of data processed daily in pathology diagnostics can be substantial. This leads to a large operational carbon footprint, and widespread implementation of DL in pathology could have considerable global warming potential^12^. Therefore, environmental sustainability should be considered already during the model development process. To tackle this, we propose an approach that allows the assessment and integration of both the performance and carbon footprint of a DL model. We introduce the ESPer score as a quantitative metric enabling consideration of ecological consequences in DL model benchmarking in addition to model performance.

The ESPer score can be calculated using any performance metric and for a single inference or including training and inferences over time. This allows selecting the most suitable model for specific tasks and situations and short- or long-term use. This is important, since for long-term use, the inference-associated CO_2_eq emissions are likely dominant, which is also true for large generative models. Using ESPer is straightforward and easy to implement. Increasingly, scientific journals encourage researchers to include CO_2_eq emissions produced by a study. ESPer could provide the next step of dissemination within the scientific community, e.g., including it in checklists such as MI CLAIM^5^. For commercial entities, ESPer could be included in legal and regulatory frameworks.

Several strategies can be used to lower carbon emissions while maintaining performance, including a reduction of the number of image tiles or selecting different tile sizes with different resolutions. In addition, model quantization and pruning^21^ using toolboxes such as EfficientBioAI^22^, which was specifically developed for biomedical imaging tasks, can be effective strategies to reduce carbon footprint. However, this was not investigated in this work, since all examined models include a component that is based on pretrained weights. ESPer can be useful to monitor the efficiency of such approaches allowing to simultaneously monitor diagnostic and environmentally sustainable performance. In our study, we showed that even in a complex classification task, data reduction strategies can be implemented to reduce the carbon footprint without compromising classification accuracy. Albeit the data reduction strategy was effective for both our tested tasks, it likely remains dataset and task-specific and should be tested during model development, for which ESPer could be a helpful metric. It is worth noting that with a large number of cases, the impact of model inference increases and mostly outweighs the impact of model training in the long run.

The range of CO_2_eq emissions can vary for different tasks and models in contrast to performance metrics which stay within the same range, typically [0–1]. We approached this by normalizing the CO_2_eq emission values in the range of tested models and introducing a non-linear component to make large variations in the exponential range more impactful. This became obvious when examining the ESPer scores of Prov-GigaPath for both tasks. For RCC task, the AUROC of Prov-GigaPath was second best after TransMIL, slightly higher than CLAM, however the ESPer score was consistently the lowest because of the high CO_2_eq emission. Similarly, for the KTX task, where Prov-GigaPath has higher AUROCs than InceptionV3 and ViT but worse ESPer scores.

ESPer also varies based on the chosen performance metrics. We used the AUROC, as this is the most commonly used performance metric in computational pathology for diagnostic classification tasks. In some settings, e.g., when the classes are not evenly distributed, the AUROC is not optimal. Therefore, we provided different ESPers for different performance metrics. If precision were to be prioritized over AUROC, for example, CLAM would emerge as the preferred method for RCC task.

Our study has several limitations. First, when calculating CO_2_eq emissions, we only focused on energy consumption and not the whole product life cycle. Thereby, additional sources of CO_2_eq emissions and other ecologically relevant aspects, such as water usage, hardware manufacturing, or shipping, were not considered. Therefore, our analyses likely underestimate the total CO_2_eq emissions. Next, while the energy consumption of the computational devices can be measured via low-level software interfaces, the actual CO_2_eq emission can only be estimated using historical data on regional carbon intensity. The carbon intensity of a region is dependent on the region’s energy mix and shows the amount of CO_2_eq emitted per kc g/kWh^23^. We did not have real-time access to the local energy mix of our region and, therefore, refer to historical data. In theory, transformation to fully renewable electricity sources or decarbonizing measures could provide a solution to the CO_2_eq emissions due to computation. Although the share of renewable energy has been steadily increasing in Germany, on average, it did not change substantially in the last few years, and in the short and even mid-term such transformation seems not likely^24^. CO_2_eq can be calculated for different regions where researchers and users need to train or use their DL models.

The aim of this study was not to tackle the challenges in ecological sustainability of the entire healthcare system. In comparison to the overall projected CO_2_eq emissions of healthcare, the absolute values from our use case within digital pathology might seem negligible. However, the focus of this study lies in providing a framework for the evaluation of performance and CO_2_eq emissions for deep learning models. Given the increasing digitization and amount of data produced in healthcare requiring an increased amount of computation, the overall impact might be larger than the concrete absolute values shown in our study, and certification of diagnostic medical devices should show a conscious effort to document efforts to reduce energy consumption, especially in high-volume applications.

Regarding data reduction strategies, we observe that the effect of such reduction methods is highly task dependent, as shown in section “Reduction strategies improving ESPer”. Although the effectiveness of these reduction strategies cannot be guaranteed, we highly recommend the exploration of such methods using ESPer to maximize CO_2_eq -reduction. We did not employ early stopping to reduce variability and to make model development more comparable.

For future works, a more comprehensive analysis of total CO_2_eq emissions is required, including CO_2_eq produced during the production cycle and supply chain of computational hardware and sourcing of raw materials. Additional to CO_2_eq emissions, water consumption as one of the most important natural resources should be included in the analysis as well. Previous works have shown that state-of-the-art LLMs require an unexpectedly large amount of water for each generated prompt response^25^. Similarly, the impact of large deep learning models in health care could be substantial as well.

In conclusion, our study proposes a potential approach to support researchers and developers in designing best-performative, but at the same time ecologically sustainable DL models. Our study should foster further development and refinement of the proposed approach, eventually leading to more ecologically responsible medical DL^26^. This aligns with the sustainable development goals 3 and 13 of the United Nations. We provide a checklist for researchers as a guide for considering sustainability during model development (Supplementary Table 1). While our study focused on pathology, the approach is application-agnostic and could potentially also be used for other medical areas.

## Methods

### Data collection

We trained on the same multi-center cohorts as in our previous study^2^. In short, one cohort derived from the Amsterdam Medical Centre (AMC) that contained 1130 biopsies (3390 whole slide images (WSI), inclusion period: 01-01-2000 till 01-06-2018 and 01-01-2019 till 31-12-2019) of kidney allograft biopsies and one cohort of kidney allograft biopsies derived from the University Medical Centre Utrecht (UMCU) that contained 717 biopsies (2151 WSI, inclusion period: 01-01-2000 till 31-12-2019) were used as the training dataset by combining both cohorts. The previously used Aachen cohort (AC) was expanded and now includes 173 biopsies (657 WSI, inclusion period: 01-01-2019 till 31-12-2022). Each case contained WSI of PAS- (periodic acid Schiff), HE- (hematoxylin and eosin), and silver stains (at least one of each stain per case). Different scanners were used to obtain the WSI: the Philips IntelliSite Ultra Fast Scanner was used in Amsterdam, which was also used for 448 cases of the UMCU cohort. The remaining 269 cases from UMCU were scanned with a Hamamatsu XR scanner. The previously used Aachen cohort (AC) was expanded and now includes 173 biopsies (657 WSI, inclusion period: 01-01-2019 till 01-01-2022). Each case contained WSI of PAS-, HE- and silver stains (at least one of each stain per case). The Aachen cohort was digitized using a Leica AT2 scanner. WSI quality control was performed manually, as described before. All cases were assigned to classes by at least two trained nephropathologists using the newest Banff classification for guidance.

Details such as exclusion and inclusion criteria, diagnosis of cases, and other patient characteristics can be found in ref. ^2^. As described previously^2^, all cases were classified into 6 classes by at least two to three experienced nephropathologists using the 2019 Banff classification: Normal, TCMR, ABMR, Mixed, Viral and Other diseases. For the specific tasks that are investigated, the classes are combined, i.e., Class Normal: Normal, Class Rejection: TCMR, ABMR and Mixed and Class Other: Viral and Other diseases. Patient characteristics and diseases included in the other diseases category can be found in the supplementary material of ref. ^2^.

For renal cell carcinoma (RCC) subtype classification, we also trained on a multi-center cohort, including the Cancer Genome Atlas (TCGA) dataset and the Aachen-RCC dataset collected at our own institute. The TCGA dataset includes three classes, clear cell renal cell carcinoma (ccRCC), papillary renal cell carcinoma (papRCC), chromophobe renal cell carcinoma (chRCC), and a total of 940 patients, with one HE-stained slide each. This was used as a training dataset. The Aachen-RCC cohort includes 289 patients (inclusion period 01-01-2012 till 31-12-2019) with one HE-stained slide per patient, digitized using a Leica AT2 scanner using the 40x objective. This cohort was used as an external testing dataset.

### Ethics declaration

Data collection and analysis in this study was performed in accordance with the Declaration of Helsinki and was approved by the local ethics.

Committee (Amsterdam 19.260; Utrecht 19.482; Aachen EK-No. 315/19). All analyses were performed retrospectively in an anonymous fashion and the need for informed consent was waived by the local ethics and privacy committee for all datasets.

### Preprocessing

To minimize noise from unrelated structures and background artifacts, the relevant regions containing kidney tissues without or with minimal artifacts were found first, either by a tissue detector algorithm or manually by a qualified professional (RDB). All WSIs were then tessellated into tiles of size 256 uM × 256 uM and saved as 224 pixel × 224 pixel images. To improve the quality of the dataset, a smoothing filter was used with a threshold of 0.15, excluding patches that were either too blurry or contained artifacts that obscured relevant structures. Additionally, white threshold filters with a threshold of 0.95 were applied during the sorting process to remove patches with insufficient information or low staining visibility.

A pretrained backbone was used for all models. In the case of InceptionV3, vision transformer, and Prov-GigaPath, the model was initialized on pretrained weights and was frozen except for the classification head.

### Deep learning models

Four different deep learning models were included in the benchmarking to represent the current state-of- the-art in MIL.

InceptionV3^16^ and vision transformer (ViT)^17^ are both patch-based architectures commonly used for image classification.

InceptionV3, a CNN based architecture, was shown by Kers et al.^2^ to outperform similar networks such as ResNet-50^27^ or EfficientNet^28^ on the kidney transplant dataset to ours. The vision transformer (ViT) published by Dosovitskiy et al. ^17^ is based on the Transformer architecture proposed by Vaswani et al.^29^ and repurposed for image classification. To take into account recent trends in foundation models, we also include Prov-GigaPath^13^ in our benchmarking. Prov-GigaPath is a whole-slide pathology foundation model trained on H&E stained slides of various cancer datasets, which has been shown to have promising performances on a variety of downstream tasks.

InceptionV3, ViT, and Prov-GigaPath were used as patch-based classifiers with a pooling-based MIL approach.

CLAM^15^ is a feature-based MIL approach using clustering and attention-layers, which achieved success in binary classification tasks such as renal cell carcinoma (RCC) subtype classification on the TCGA dataset.

TransMIL^14^ is also a feature-based MIL approach which utilizes a hybrid architecture with Transformer based self-attention mechanisms. TransMIL compares favorably against CLAM in various benchmarks^30,31^.

### Performance metric

Five performance metrics were reported, namely area under the receiver operating characteristics curve (AUROC), balanced accuracy, precision, recall and F1-index. The mean AUROC is calculated as a macro averaged over all class scores. These metrics represent the most widely reported metrics for classification tasks in deep learning and they were chosen to allow easy adaptation for existing methodology.

### CO_2_-emission calculation

The energy consumption of each experiment was measured using the publicly available experiment-impact-tracker package (version 0.1.9) with specific configurations of a Python environment with the following libraries: cuda-toolkit (11.3.0), Python (3.9.16), Pytorch (2.1.0), openslide-python (1.2.0); on Linux servers (Ubuntu 20.04.6 LTS). All measurements were conducted on a Nvidia DGX-1 GPU server with Tesla V100 architecture with a PUE (power usage effectiveness) of 1.58 for our local server room. For reference, one idle GPU consumes 0.0034 kWh under the same experiment conditions.

The CO_2_eq was calculated by multiplying the energy consumption with the yearly averaged carbon intensity of the geographical region of interest. The carbon intensity shows the amount of CO_2_eq emitted per kWh of energy produced in a region and is measured in g/kWh^23^.

The energy consumption for training was measured over 300 epochs for all models and the energy consumption for inference on one slide was measured by averaging the measurements over thousand slides.

While it is common practice to extract image features in a separate step for simple convenience, we perform feature extraction during training- and inference-time for feature-based MIL models to make a fair comparison to the image-based models.

The scanning and storage process was not included in the measurements.

### Environmentally sustainable performance score

We combine performance metrics and the carbon equivalent emissions CO_2_eq into a singular metric, the environmentally sustainable performance (ESPer) score, which formulated for inference (iESPer) is defined as seen in Fig. 1b.

Where ${iESPe}{r}_{i,{Perf}}$ is the iESPer score for model $i$ in the comparison series and performance metric ${Perf}$, ${M}_{i,{Perf}}\in [\mathrm{0,1}]$ is the measured metric for model $i$, $w\in [\mathrm{0,1}]$ is the weighting factor, ${{CO}}_{2}{{eq}}_{i,\inf }$ is the CO_2_eq produced by model $i$ during inference and $X{\prime}$ is the range normalization operation for $X$. The square of ${M}_{i,{Perf}}$ is taken to reward highly accurate models. Any performance metric can be used for ${M}_{i,{Perf}}$ as long as the metric is within the defined range of [0,1]. Choosing $w=1$ represents the default configuration, $w < 1$ decreases the weight of CO_2_eq and $w=0$ weighs performance only. Choosing a suitable weighting factor is highly task-specific and we leave it up to the user to decide. For all experiments in this study $w$ is set to one. The lowest and highest emissions in the series are used as lower and higher bounds for normalization and an exponential function is introduced to penalize high CO_2_ emissions and avoid zero-division.

When using ESPer for future usage projections, the CO_2_eq emissions for inference ${{CO}}_{2}{{eq}}_{i,\inf }$ are multiplied by a projected number of usage ${n}_{{usage}}$ for the estimated time frame and added to the CO_2_eq of the training process ${{CO}}_{2}{{eq}}_{i,{train}}$, as shown in Fig. 1c.

## Supplementary information

Supplementary Information

## Data Availability

The raw whole slide image data are available under restricted access for privacy protection reasons, access can be obtained by directly contacting Peter Boor, Institute of Pathology, RWTH Aachen University Clinic, Aachen, Germany, pboor@ukaachen.de (for the Aachen_RCC and Aachen_KTX datasets). In general, the requests will be evaluated within 4 weeks based on institutional policies. The prerequisite for exchanging data or models is a data transfer agreement, approved by the legal departments of the requesting researcher and by all legal departments of the institutions that provided data for the study, as well as an ethics clearance. The public image and clinical data used in this study are available in the TCGA database (https://www.cancer.gov/ccg/research/genome-sequencing/tcga).

## Code availability

The code is available at https://git-ce.rwth-aachen.de/labooratory-ai/esper.
