# Supplementary material for: Ecologically sustainable benchmarking of AI models for histopathology
Source: NPJ Digit Med. 2024 Dec 24;7:378. doi: 10.1038/s41746-024-01397-x (PMC11668825; doi:10.1038/s41746-024-01397-x)
Supplement: Supplementary file 1 — Supplementary Information [file 41746_2024_1397_MOESM1_ESM.pdf]

Supplementary Material to

Ecologically sustainable benchmarking of AI models for histopathology

Yu-Chia Lan[1], Martin Strauch[1], Pourya Pilva[1], Nikolas E J Schmitz[1], Alireza Vafaei Sadr[1,2], Leon Niggemeier[1], Huong Quynh Nguyen[1], David L. Hölscher[1,3], Tri Q Nguyen[4], Jesper Kers[5,6], Roman D. Bülow[1,*], Peter Boor[1,3,*,#]

1 Institute of Pathology, RWTH Aachen University Hospital, Aachen, Germany

2 Department of Public Health Sciences, College of Medicine, The Pennsylvania State University, Hershey, USA

3 Department of Nephrology and Clinical Immunology, RWTH Aachen University Hospital, Aachen, Germany

4 Department of Pathology, University Medical Centre Utrecht, Utrecht, The Netherlands

5 Department of Pathology, Amsterdam UMC, University of Amsterdam, Amsterdam, The Netherlands

6 Department of Pathology, Leiden Transplant Center, Leiden University Medical Center, Leiden, The Netherlands

*These authors contributed equally

# Address correspondence to:
Prof. Peter Boor
Institute of Pathology
RWTH Aachen University Hospital
Pauwelsstraße 30
52074 Aachen
pboor@ukaachen.de

Supplementary Tables

Supplementary Table 1

Proposed sustainability checklist for use in medical imaging AI studies.

Before Benchmarking			
	Completed (page number of manuscript)		Not completed because:
Models have been surveyed and filtered by performance and model size	☐		
Availability of suitable pre-trained backbone models has been assessed	☐		
Most suitable image resolutions have been surveyed in preliminary tests	☐		
External Validation			
iESPer and fpESPer scores were calculated to determine the most ecologically sustainable method	☐		
Data reduction strategies for inference were investigated	☐		
Model pruning, compression and knowledge distillation were investigated	☐		
Estimated CO_2eq emissions of model training for the study are reported	☐		

Supplementary Table 2

The difference in iESPer scores for model performance at different epochs was investigated. TransMIL was trained on RCC task and early stopping using validation accuracy during training was applied. The table shows the CO2eq emissions and iESPer scores of the model during different epochs.

Epochs	AUROC	CO_2eq /Slide (kg)	iESPer
213	0.976	2.89	0.953
234	0.971	3.17	0.741
244	0.970	3.31	0.659
295	0.977	4.00	0.372
300	0.978	4.07	0.352

Supplementary Figures

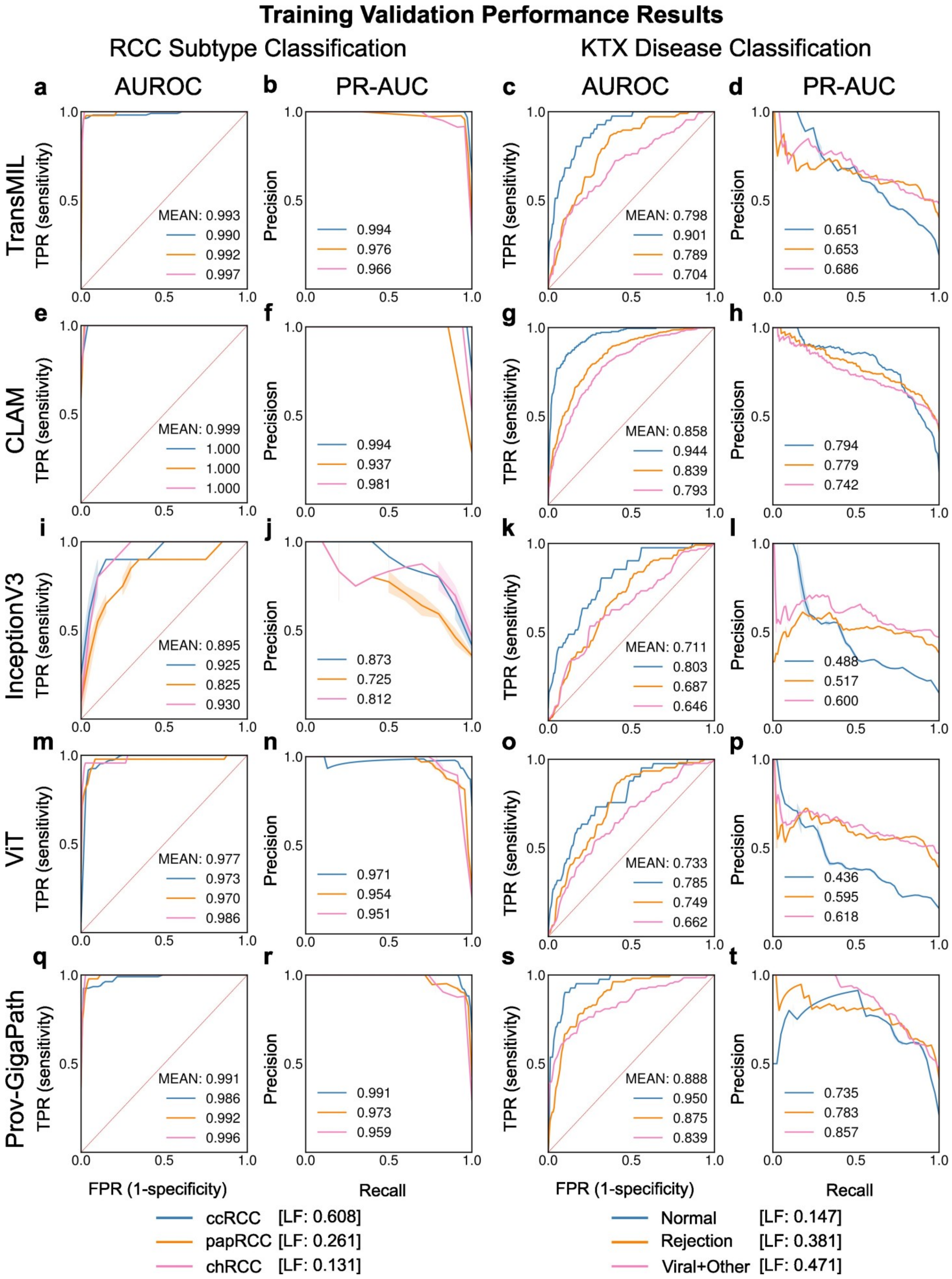

Supplementary Figure 1: Performance results for RCC subtype classification and KTX disease classification on training validation set.

This figure shows the performance results of the models TransMIL, CLAM, InceptionV3 and ViT on the training validation dataset for RCC (n=177) and KTX (n=525). (a), (e), (i), (m) and (w) show the AUROC and (b), (f), (j), (n) and (r) show the AUPRC for all models for RCC, respectively. (c), (g), (k), (o) and (d) show the AUROC and (f), (h), (l), (p) and (t) show the AUPRC for all models for KTX, respectively. LF = label frequency of the corresponding class, TPR = True Positive Rate, FPR = False Positive Rate, AUROC = Area Under the Receiver Operating Characteristics Curve, PR-AUC = Precision-Recall Area Under the Curve, RCC = Renal Cell Carcinoma, ccRCC = Clear Cell Renal Cell Carcinoma, papRCC = Papillary Renal Cell Carcinoma, chRCC = Chromophobe Renal Cell Carcinoma.

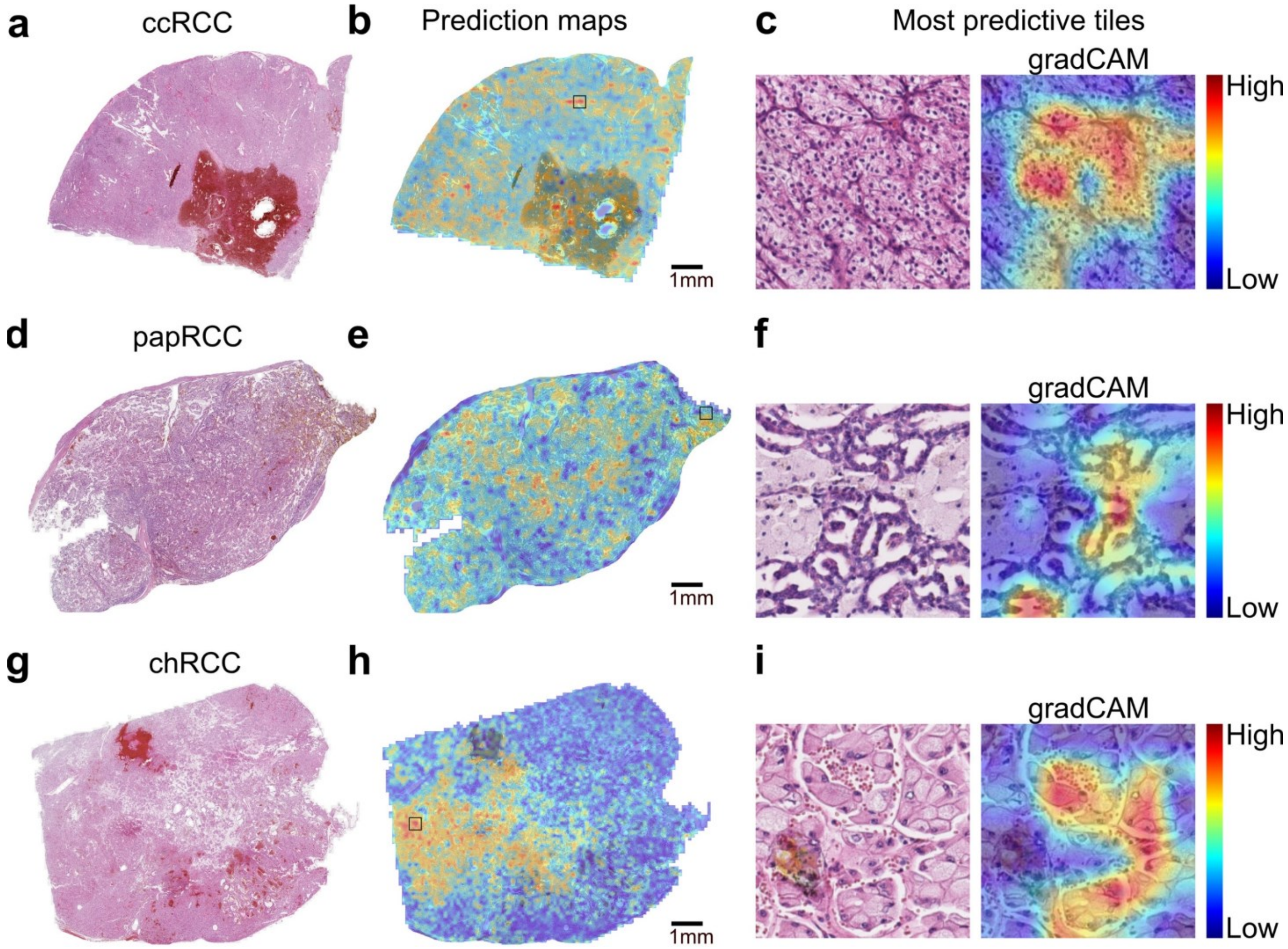

Supplementary Figure 2: TransMIL RCC visualization of predictions on external Aachen-RCC cohort

(a), (b), (e) and (h) show patch-level GradCAM visualizations of highly predictive patches (left) and pixel-level GradCAM visualizations of patches with high prediction scores (right), for each class, respectively. (c), (f), and (i) show detailed pixel-level GradCAM visualizations for each parent slide. Tile edge length is 256µm. GradCAM = Gradient-weighted Class Activation Mapping, ccRCC = Clear Cell Renal Cell Carcinoma, papRCC = Papillary Renal Cell Carcinoma, chRCC = Chromophobe Renal Cell Carcinoma.

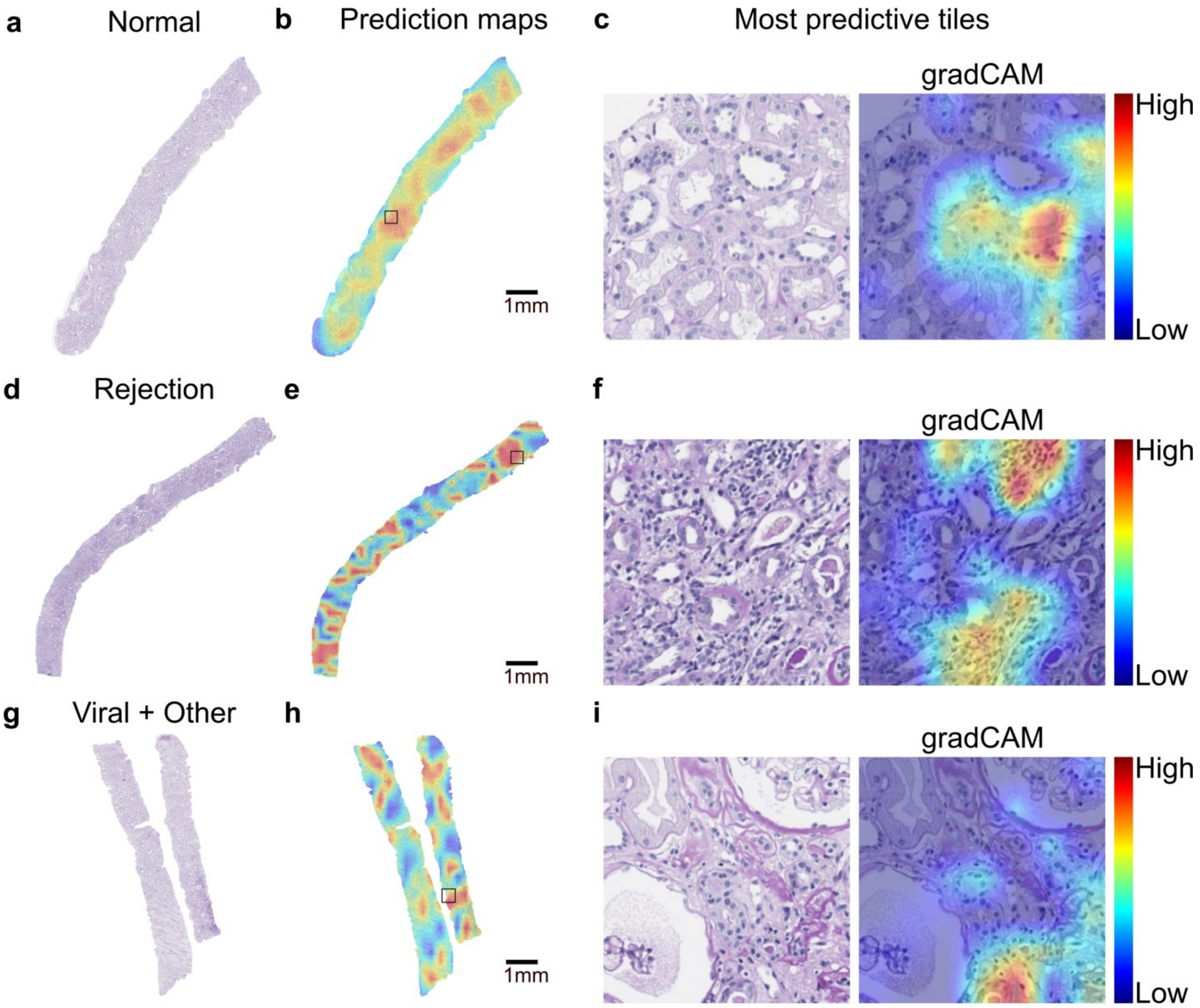

Supplementary Figure 3: TransMIL KTX visualization of predictions on external Aachen-KTX cohort

Patient-level receiver operator characteristic curves for the three classes Normal, Rejection and Other are shown in (a), (d) and (g), respectively. (b), (e) and (h) show patch-level GradCAM visualizations of highly predictive patches (left) and pixel-level GradCAM visualizations of patches with high prediction scores (right), for each class, respectively. (c), (f), and (i) show detailed pixel-level GradCAM visualizations for each parent slide. Tile edge length is 256µm. GradCAM = Gradient-weighted Class Activation Mapping.

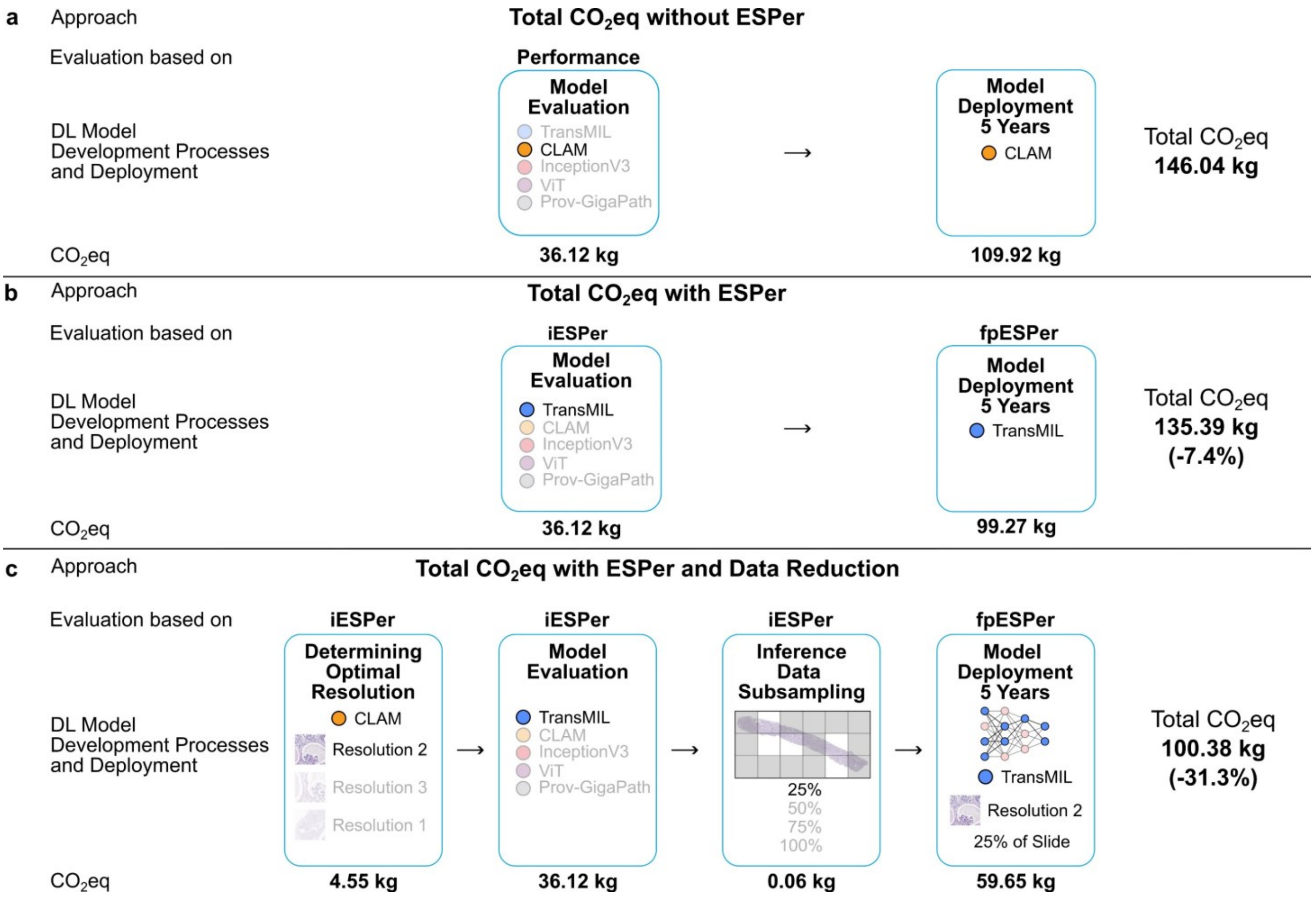

Supplementary Figure 4: Different scenarios of deep learning (DL) model development with and without using ESPer for RCC subtype classification.

(a) CO_2eq emissions when evaluating models without using ESPer. The best model is chosen only based on performance. (b) CO_2eq emissions when evaluating models based on ESPer. The best model is chosen based on iESPer, leading to a decrease of CO_2eq by 7.4% when compared to scenario (a). (c) CO_2eq produced when using ESPer and data reduction methods. First, the most suitable image resolution for the task is determined on a subset of the training data using iESPer. Second, all candidate models are evaluated using the best-suited image resolution and finding the best model using iESPer. Third, the optimal fraction of inference input data needed is determined based on validation data with iESPer. In comparison to (a), approach (c) leads to a decrease of CO_2eq by 31.3%. The calculations for five five-year inferences were based on the number of RCC cases (n=431288) reported worldwide in 2022.

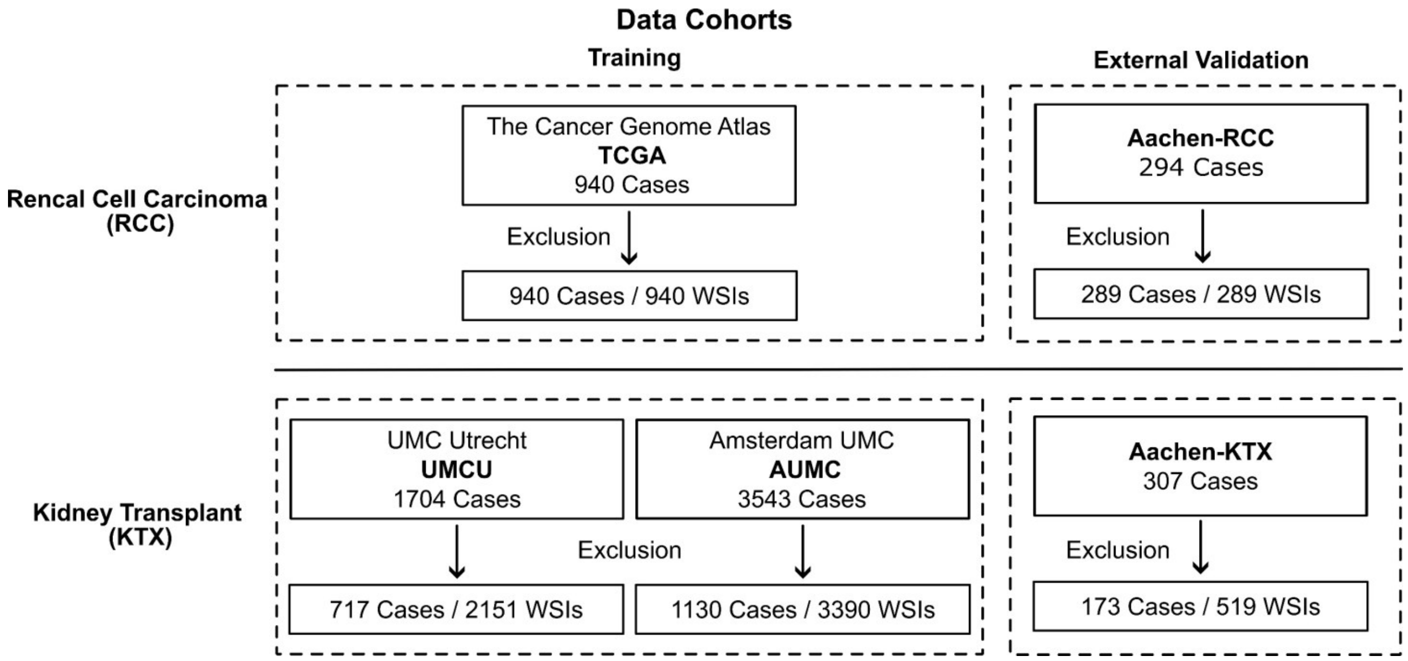

Supplementary Figure 5: Data Cohorts

This figure shows the data cohorts collected for the tasks examined in the study.

For Renal Cell Carcinoma, 940 cases (940 WSIs) from the TCGA cohort were used for training and 289 cases (289 WSIs) from our own Aachen-RCC cohort were used for external validation.

For Kidney Transplant, the UMCU cohort with 717 cases (2151 WSIs) and the AUMC cohort with 1130 cases (3390 WSIs) were used for training. Our own Aachen-KTX cohort with 173 cases (519 WSIs) was used for external validation.
